# Phosphorylation of Kif26b Promotes Its Polyubiquitination and Subsequent Proteasomal Degradation during Kidney Development

**DOI:** 10.1371/journal.pone.0039714

**Published:** 2012-06-29

**Authors:** Takeshi Terabayashi, Masaji Sakaguchi, Kaori Shinmyozu, Toshio Ohshima, Ai Johjima, Teru Ogura, Hiroaki Miki, Ryuichi Nishinakamura

**Affiliations:** 1 Department of Kidney Development, Institute of Molecular Embryology and Genetics, Kumamoto University, Kumamoto, Japan; 2 The Global COE “Cell Fate Regulation Research and Education Unit,” Kumamoto University, Honjo, Kumamoto, Japan; 3 Proteomics Laboratory, RIKEN Center for Developmental Biology, Kobe, Hyogo, Japan; 4 Department of Life Science and Medical Bio-Science, Waseda University, Tokyo, Japan; 5 Department of Molecular Cell Biology, Institute of Molecular Embryology and Genetics, Kumamoto University, Kumamoto, Japan; 6 Department of Cellular Regulation, Research Institute for Microbial Diseases, Osaka University, Osaka, Japan; The Hong Kong University of Science and Technology, Hong Kong

## Abstract

Kif26b, a member of the kinesin superfamily proteins (KIFs), is essential for kidney development. *Kif26b* expression is restricted to the metanephric mesenchyme, and its transcription is regulated by a zinc finger transcriptional regulator Sall1. However, the mechanism(s) by which Kif26b protein is regulated remain unknown. Here, we demonstrate phosphorylation and subsequent polyubiquitination of Kif26b in the developing kidney. We find that Kif26b interacts with an E3 ubiquitin ligase, neural precursor cell expressed developmentally down-regulated protein 4 (Nedd4) in developing kidney. Phosphorylation of Kif26b at Thr-1859 and Ser-1962 by the cyclin-dependent kinases (CDKs) enhances the interaction of Kif26b with Nedd4. Nedd4 polyubiquitinates Kif26b and thereby promotes degradation of Kif26b via the ubiquitin-proteasome pathway. Furthermore, Kif26b lacks ATPase activity but does associate with microtubules. Nocodazole treatment not only disrupts the localization of Kif26b to microtubules but also promotes phosphorylation and polyubiquitination of Kif26b. These results suggest that the function of Kif26b is microtubule-based and that Kif26b degradation in the metanephric mesenchyme via the ubiquitin-proteasome pathway may be important for proper kidney development.

## Introduction

The kidney is composed of minimum units called nephrons, which maintain an appropriate homeostatic balance of water and salt levels and remove nitrogenous metabolic waste products. The tubular epithelial network of the nephron originates from two different tissues, the ureteric bud and the metanephric mesenchyme, that form the collecting duct system and the renal tubules, respectively [1], [2]. The signals from the metanephric mesenchyme, such as glial cell line-derived neurotrophic factor (GDNF), induce sprouting of the ureteric bud from the caudal region of the Wolffian duct and invasion of the ureteric buds into the metanephric mesenchyme. Wnt9b secreted from the ureteric buds induces Wnt4 expression in the mesenchyme [3], and Wnt4 induces the pre-tubular aggregates of the condensed mesenchyme beneath the ureteric bud tips to form renal vesicles in a cell-autonomous manner [4]. Renal vesicles differentiate into each segment of the nephron, including the glomerulus, proximal tubule, loop of Henle, and distal tubule, to eventually form functional nephrons [1], [2].

The kinesin superfamily proteins (KIFs) are known to be important molecular motors that are involved in the microtubule- and ATP-dependent transport of various cargos, including membranous organelles, protein complexes, and mRNAs, to specific destinations [5]. Accumulating evidence demonstrates the importance of KIFs in the regulation of many physiological events, including higher brain function, tumor suppression, and developmental patterning. Kif26b was originally identified by a database search of the mouse genome for DNA sequences that contained a motif similar to the kinesin motor domain [6]. Kif26b is classified to the Kinesin-11 family along with Kif26a, an unconventional kinesin that lacks microtubule-based motility [7]. Human KIF26A does not contain the conserved amino acid sequences that are required for motor activity but retains the microtubule-associating ability as well as other conserved KIFs. Smy1p, a Kinesin-11 family member from *Saccharomyces cerevisiae*, is thought not to be motile, especially along microtubules, due to the deviance in both a catalytic pocket for ATP hydrolysis and the microtubule-binding sites [8]. These reports suggest that Kif26b also does not function as a microtubule-based motor. Therefore, the biochemical and cellular functions of Kif26b remain to be clarified.

We recently reported that *Kif26b*-knockout mice exhibit kidney agenesis or hypoplasia [9]. In *Kif26b*-null embryos, the ureteric buds elongate and migrate in proximity to, but do not invade, the metanephric mesenchyme. This defect is attributable to impaired maintenance of *Gdnf*, which is regulated by α8β1 integrin signaling [10]. Indeed, *Kif26b*-null mesenchyme does not condense around the ureteric buds, indicating that the localization of the integrin α8 subunit is disrupted. Furthermore, transcription of *Kif26b* is regulated by Sall1, a zinc finger transcription factor that has been reported to be crucial for kidney development [11]. The promoter region of *Kif26b* contains tandem Sall1-binding consensus sequences, and binding of Sall1 to these sites enhances *Kif26b*-transcription. In the developing kidney, *Kif26b* is expressed in the undifferentiated metanephric mesenchyme but is rapidly downregulated after renal vesicle formation. While our study revealed the regulatory mechanism of *Kif26b* transcription, the regulation of Kif26b protein during kidney development remains to be elucidated.

In the present study, we identify Nedd4, a HECT E3 ubiquitin ligase, as a Kif26b-interacting partner. Nedd4 polyubiquitinates Kif26b and thus targets it for degradation via the ubiquitin-proteasome pathway. We also show that phosphorylation of Kif26b by CDKs is important for its interaction with Nedd4. Interestingly, disruption of microtubules by nocodazole triggers phosphorylation and polyubiquitination of Kif26b. These findings suggest that the degradation of Kif26b is important for kidney development.

## Materials and Methods

### Reagents and Antibodies

Roscovitine, a selective CDK inhibitor, was purchased from Biomol (Plymouth Meeting, PA). The proteasome inhibitor MG132, the MEK1/2 inhibitor U0126, and nocodazole were from Peptide Institute (Osaka, Japan), Wako (Osaka, Japan), and Sigma-Aldrich (St. Louis, MO), respectively. Anti-Kif26b rabbit polyclonal antibody was previously described [9]. Antibodies against phosphorylated Kif26b (anti-phospho-Thr1859 and anti-phospho-Ser1962 Kif26b antibodies) were generated by immunization of rabbits with phosphorylated peptide (phospho-Thr1859; CYSKIpTPPRKP (1855–1864) and phospho-Ser1962; CLDTPpSPVRKT (1958–1967)) conjugated to KLH and the resulting sera then affinity-purified. The following commercially available antibodies were also used: rabbit polyclonal anti-c-Myc antibody, rabbit polyclonal anti-Nedd4 antibody and mouse monoclonal anti-ubiquitin antibody (Santa Cruz Biotechnology, Santa Cruz, CA), rabbit polyclonal anti-phospho CDK/MAPK substrate (phospho-Ser) antibody, mouse monoclonal anti-phospho CDK/MAPK substrate (phospho-Thr) antibody, and rabbit polyclonal anti-CDK5 antibody (Cell Signaling, Danvers, MA), rabbit polyclonal anti-FLAG antibody and mouse monoclonal anti-β-tubulin antibody (Sigma-Aldrich), and Alexa Fluor 488 goat anti-rabbit IgG and Alexa Fluor 546 goat anti-mouse IgG (Invitrogen, Carlsbad, CA).

### Plasmids

The full-length Kif26b construct and the Kif26b-ΔC and Kif26b-C deletion constructs were previously described [9]. The other Kif26b deletion constructs were generated by digestion by appropriate restriction enzymes or by PCR. The amino acid residue numbers of the deletion constructs are as follows: Motor (1–798) and ΔMotor (799–2112). The full-length GAKIN/KIF13B construct was previously described [12]. The cDNA for full-length mouse Nedd4 was kindly gifted by Dr. Shinji Masui at the Center for iPS Cell Research and Application, Kyoto University. The Nedd4 deletion constructs were generated by PCR with appropriate primers. The amino acid residue numbers of the deletion constructs are as follows: C2 (1–249), ΔC2 (250–886), WW (250–549), and HECT (550–886). Point mutations in Kif26b (T1859A, S1962A, and T1859A/S1962A) and Nedd4 CS (the catalytically inactive C854S mutation) were introduced with a site-directed mutagenesis kit (Agilent Technologies, Santa Clara, CA). All DNA fragments were sequenced for verification.

### Cell Culture and Transfection

HEK293, COS-7, and HeLa (American Type Culture Collection) cells were maintained in DMEM supplemented with 10% fetal bovine serum. Cells were transfected using Lipofectamine 2000 (Invitrogen) according to the manufacturer’s instructions. For organ culture, kidneys dissected from embryonic day (E) 14.5 embryos were cultured as previously described [13]. MG132 was used at a final concentration of 20 µM.

### RNA Interference

Duplex siRNAs for human Nedd4 were purchased from Invitrogen. The siRNA nucleotide sequences were as follows: Nedd4-siRNA#1, sense (5′-UAUUGGUGACAACUAUUUCUGAUCC-3′) and antisense (5′-GGAUCAGAAAUAGUUGUCACCAAUA-3′), Nedd4-siRNA#2, sense (5′-UUCAAUUGCCAUCUGAAGUUUAUCC-3′) and antisense (5′-GGAUAAACUUCAGAUGGCAAUUGAA-3′), and negative control, sense (5′-AAAUGGCGUCGCGUUCCUUUAGAGG-3′) and antisense (5′-CCUCUAAAGGAACGCGACGCCAUUU-3′).

### Cell Lysis, Immunoprecipitation, and Immunoblotting

Cells were harvested with lysis buffer (50 mM Tris-HCl (pH 7.5), 100 mM NaCl, 10% glycerol, 5 mM EDTA, 0.5% Triton X-100, 0.1 mM phenylmethylsulfonyl fluoride (PMSF), and phosphatase inhibitor cocktail (Thermo, Waltham, MA). For analysis of ubiquitinated proteins, SDS was added to a concentration of 0.1%. Lysates were centrifuged at 17,500 × *g* at 4°C, and the supernatants were incubated with the appropriate antibodies and protein G-Sepharose beads (Pierce, Rockford, IL) for 2 h at 4°C. Immunoprecipitates were washed four times with lysis buffer, separated by SDS-PAGE, and transferred to a polyvinylidene difluoride membrane. Immunoblotting analyses were performed as previously described [14] with a minor modification. In brief, membranes were blocked with 10% fat-free dry milk in phosphate-buffered saline or 5% BSA in Tris-buffered saline with 0.05% Tween 20 and incubated with primary antibodies and then with alkaline phosphatase-conjugated anti-mouse IgG or anti-rabbit IgG (Promega, Madison, WI).

### Immunohistochemistry and *in situ* Hybridization

E14.5 mouse embryos were fixed in 10% formalin and processed for paraffin-embedded sectioning. Immunohistochemistry and *in situ* hybridization and were performed using an automated Discovery System (Roche, Basel Switzerland) according to the manufacturer’s protocols. A cDNA fragment of *Nedd4* (1648–2664 bp) spanning the open reading frame was used as a riboprobe. All experimental procedures and protocols for animal studies were approved by the Committee on Animal Research of Kumamoto University (B23-179).

### Immunofluorescence Analyses

Immunofluorescence analysis was performed as previously described [15]. For tubulin staining, cells grown on cover slips were fixed with methanol for 3 min on ice and rehydrated by three times 5-min washes with phosphate-buffered saline (PBS). Cells were blocked for 30 min with 2% BSA in PBS, incubated for 1 h with the primary antibody diluted in blocking buffer. After three times 5-min washes with PBS, cells were incubated for 30 min with appropriate secondary antibodies diluted in blocking buffer. Coverslips were then washed with PBS and mounted, and immunofluorescence was observed using a confocal scanning laser microscope (FLUOVIEW FV1000; Olympus, Tokyo, Japan). Images were processed with Adobe Photoshop software.

### Microtubule-binding Assay

FLAG-Kif26b or FLAG-GAKIN/KIF13B was expressed in COS-7 cells. Cells were lysed with PHEM buffer (50 mM Pipes, 50 mM HEPES (pH 7.2), 10 mM EGTA, 5 mM MgCl_2_, and 1% Triton X-100) containing complete protease inhibitor (Roche) and centrifuged at 100,000 × *g* for 30 min at 4°C. The lysates were supplemented with 0.2 mg/ml of a taxol-stabilized microtubule solution (purified from porcine brain as previously described [16]) incubated at room temperature for 30 min in the presence of 20 µM taxol and 2 mM AMP-PNP, and centrifuged at 100,000 × *g* for 15 min at 20°C. The resulting supernatant and pellet were used as S1 and P1, respectively. The P1 fraction was resuspended in PHEM containing 20 µM taxol and 10 mM ATP, incubated at room temperature for 30 min, and centrifuged at 100,000 × *g* for 15 min at 20°C. The resulting supernatant and pellet from the second centrifugation were used as S2 and P2, respectively.

### *In vitro* Kinase Assay

The *in vitro* kinase assay was performed as previously described with a minor modification [15]. In brief, a recombinant C-terminal tail fragment of Kif26b (Kif26b-C) expressed in *Escherichia coli* (BL21) with an N-terminal glutathione *S*-transferase (GST) tag was purified with Glutathione Sepharose 4B (GE Healthcare, Buckinghamshire, United Kingdom). CDK1/cyclin B, CDK2/cyclin A, CDK5/p25 and CDK6/cyclin D3 were purchased from Millipore. Kinase activity was assayed in 50 mM Tris-HCl (pH 7.5), 5 mM MgCl_2_, 2 mM EGTA, 0.5 mM PMSF, and 0.5 mM DTT for 30 min at 30°C in the presence of 0.1 mM ATP.

### *In vitro* Ubiquitination Assay

*In vitro* ubiquitination assays for Kif26b proteins were performed as previously described [17] with the following minor modifications. The FLAG-Kif26b used as a substrate was ectopically expressed in HEK293 cells and purified by anti-FLAG immunoprecipitation. The precipitates were incubated for 1 h at 37°C with 2 µg of purified GST-Nedd4 protein, 12 µg ubiquitin (Sigma-Aldrich), 100 ng E1 (His-UBE1, Boston Biochem, Cambridge, MA), and 1 µg E2 (His-Ubc7, Boston Biochem). The reactions were washed three times with lysis buffer and then analyzed by SDS-PAGE followed by immunoblotting.

## Results

### Nedd4 is a Novel Interaction Partner of Kif26b

**Figure 1:**
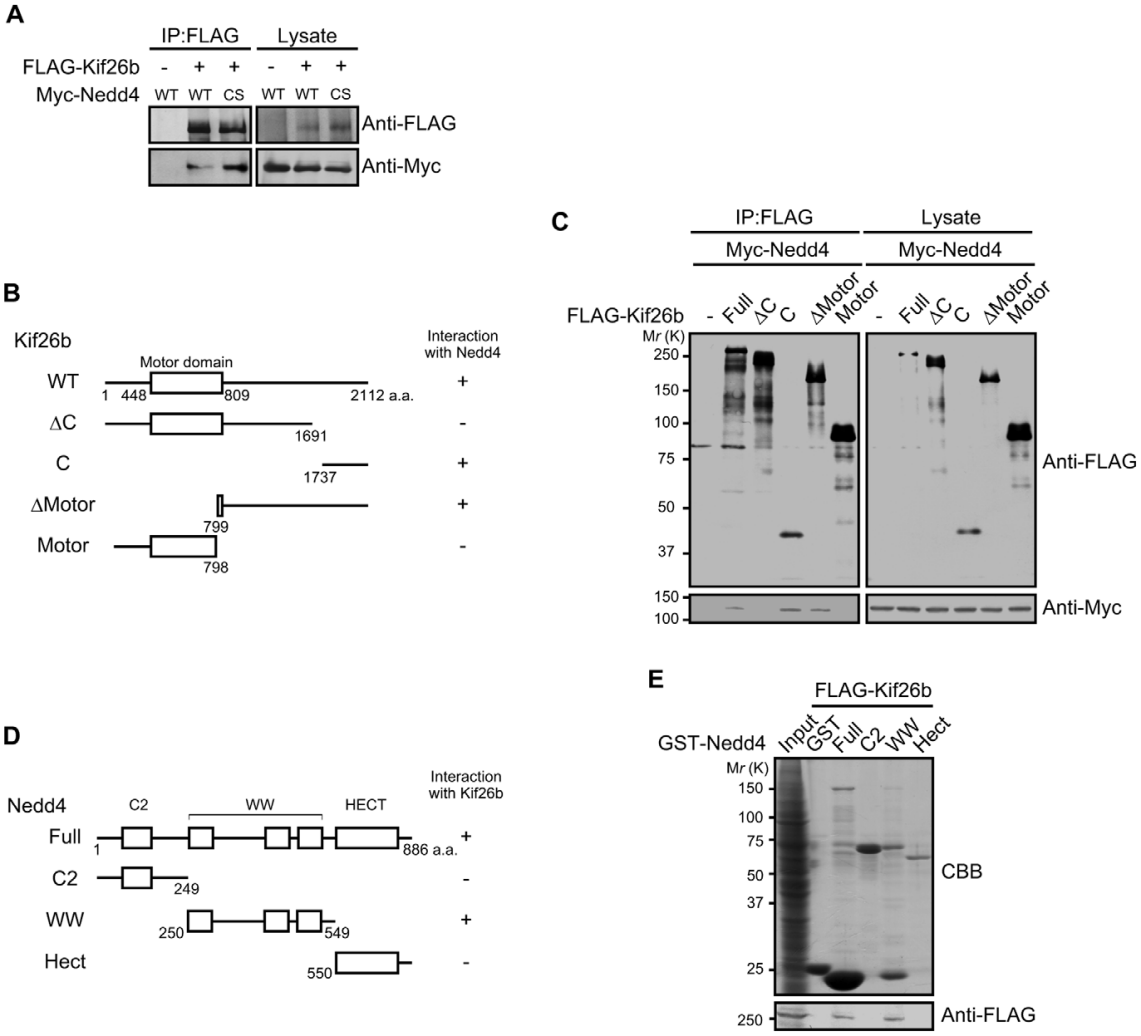
Identification of Nedd4 as a Kif26b-interacting protein. *A*. HEK293 cells were transfected with the indicated constructs. FLAG-tagged proteins were immunoprecipitated from the lysates with anti-FLAG beads. The precipitates were analyzed by immunoblotting with the indicated antibodies. *B*. Schematic diagrams of the Kif26b deletion constructs. Interaction with Nedd4 is summarized on the *right* of each construct: +, positive; −, negative. *C*. HEK293 cells were transfected with the indicated constructs. FLAG-tagged proteins were immunoprecipitated from the lysates with anti-FLAG beads. The precipitates were analyzed by immunoblotting with the indicated antibodies. *D*. Schematic diagrams of the Nedd4 deletion constructs. Interaction with Kif26b is summarized on the right of each construct: +, positive; −, negative. *E*. HEK293 cells were transfected with FLAG-Kif26b-expressing plasmids. The lysates were incubated with purified GST or GST-tagged Nedd4 deletions. The glutathione bead precipitates were analyzed by immunoblotting with the indicated antibodies.

In our previous report, we searched for proteins that interact with Kif26b in the developing kidney and found several, including nonmuscle myosin heavy chain type IIB, that were pulled down by a purified GST-tagged C-terminal tail fragment of Kif26b [9]. We analyzed the precipitated proteins by mass spectrometry and identified an approximately 120 kDa protein as Nedd4, a HECT E3 ubiquitin ligase (Figure S1). To examine the interaction between Kif26b and Nedd4, we performed overexpression and immunoprecipitation analyses. These showed obvious complex formation between Kif26b and wild type (WT) Nedd4 (Figure 1A). Interestingly, the catalytically inactivated mutant of Nedd4 (Nedd4 CS) showed a stronger affinity against Kif26b, suggesting Kif26b as a potential substrate for a HECT domain ubiquitin ligase.

We next investigated which region of Kif26b was involved in the interaction with Nedd4. We expressed Myc-tagged Nedd4 and FLAG-tagged full-length or partial Kif26b constructs (Figure 1B) in HEK293 cells and immunoprecipitated the FLAG-tagged proteins from the cell lysates with anti-FLAG beads. Among the Kif26b deletion mutants, ΔMotor and C interacted with Myc-Nedd4, suggesting that the C-terminal tail region of Kif26b is required for the interaction with Nedd4 (Figure 1C). We next constructed Nedd4 deletion mutants (Figure 1D) and performed pull-down assay to test their abilities to associate with Kif26b. As shown in Figure 1E, the WW domains of Nedd4 mediated its interaction with Kif26b. As the WW domains of Nedd4 have been implicated in substrate recognition [18], these results suggested that Nedd4 might ubiquitinate Kif26b.

### Kif26b is Phosphorylated by CDKs at Thr-1859 and Ser-1962

As shown above, the WW domains of Nedd4 interacted with the C-terminal tail region of Kif26b. The WW domains have been grouped into four classes according to their ligand preferences [19]. The WW domains of Nedd4 belong to Class-I WW domains, which bind to proteins containing PPxY (PY) motifs or phosphoserine/phosphothreonine residues [20]–[22]. As Kif26b does not contain PY motifs, we examined whether phosphorylation of Kif26b affected its interaction with Nedd4.

**Figure 2:**
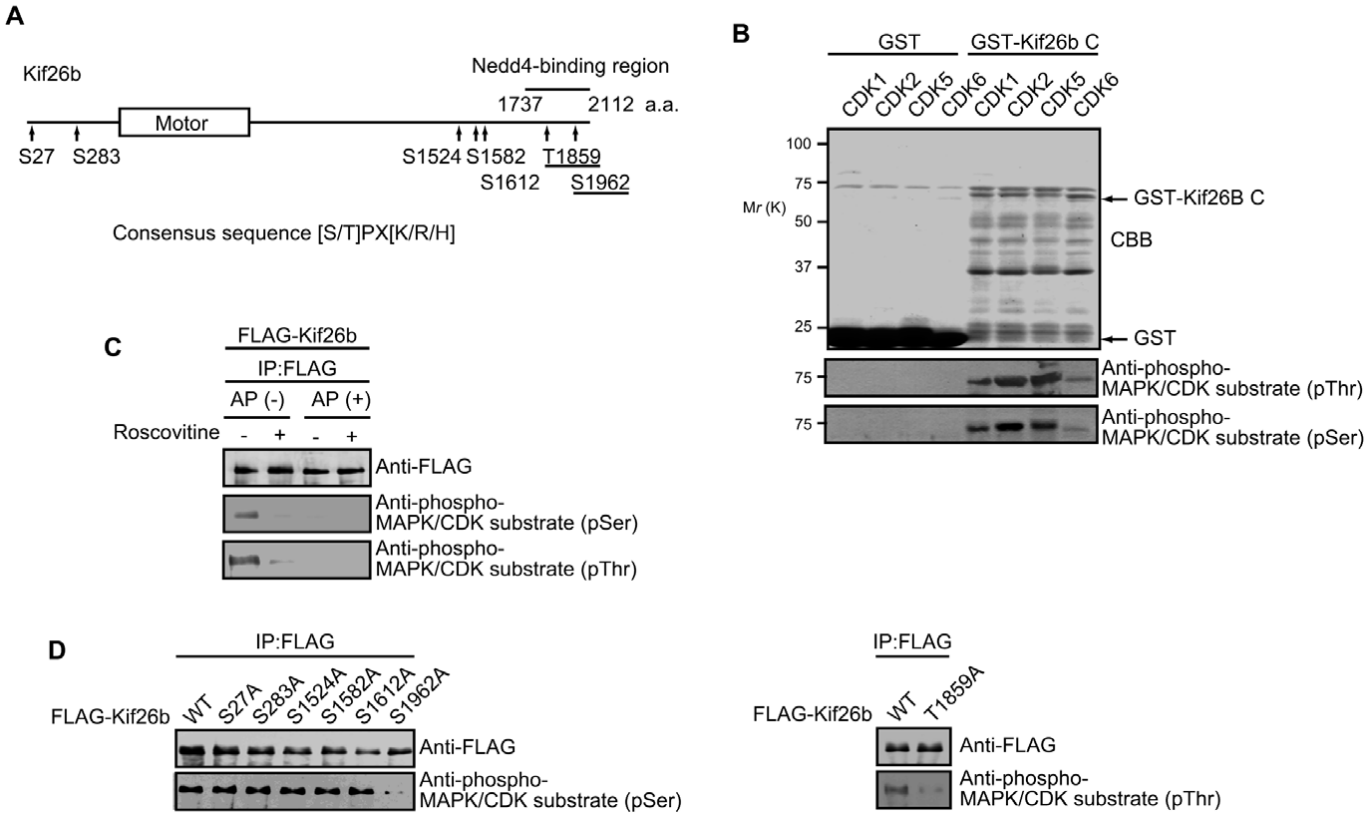
CDKs phosphorylate Thr-1859 and Ser-1962 on Kif26b. *A.* Schematic diagram of the amino acids in Kif26b potentially phosphorylated by CDK5. The C-terminal tail region of Kif26b that interacts with Nedd4 is indicated by the bar. Underlined amino acids indicate CDK5 phosphorylation sites. *B.* GST or GST-Kif26b-C was incubated with or without recombinant His-tagged CDK1, CDK2, CDK5 or CDK6 and a kinase assay was performed. Proteins were separated by SDS-PAGE and detected by Coomassie Brilliant Blue (CBB) staining or immunoblotting with the indicated antibodies. *C*. HEK293 cells were transfected with a FLAG-Kif26b-expressing plasmid. At 48 hrs post-transfection, the cells were treated for 6 h with DMSO (-) or Roscovitine (+; 20 µM). FLAG-tagged proteins were immunoprecipitated from the cell lysates with anti-FLAG beads. Precipitates were then treated with or without calf intestine-derived alkaline phosphatase (AP) at 37°C for 1 h and analyzed by immunoblotting with the indicated antibodies. *D*. HEK293 cells were transfected with WT or substitution mutant FLAG-Kif26b-expressing plasmids. The FLAG-tagged proteins were immunoprecipitated from the cell lysates with anti-FLAG beads. The precipitates were analyzed by immunoblotting with the indicated antibodies.

We first addressed whether the C-terminal tail region of Kif26b could be phosphorylated. We searched the C-terminal tail region of Kif26b for protein kinase consensus sequences and found that this region harbored two potential CDK phosphorylation sites (Figure 2A). Analyses of combinational peptide libraries have determined the consensus sequence for CDK phosphorylation to be a Ser (S)- or Thr (T)-Pro (P) motif followed by a basic amino acid (Arg (R), Lys (K), or His (H)) ([S/T]Px[R/K/H]) [23].

To examine whether CDKs could phosphorylate Kif26b, we performed an *in vitro* kinase assay using purified active CDK proteins. We found that CDK2 and CDK5 prominently phosphorylated GST-Kif26b C (Figure 2B). CDK1 showed potent but weaker kinase activity against Kif26b, but the activity of CDK6 was low. These results indicate that Kif26b is phosphorylated by CDKs, mainly CDK2 and CDK5. We next confirmed phosphorylation of Kif26b in cultured cells. FLAG-Kif26b was expressed in HEK293 cells, immunoprecipitated with anti-FLAG beads, and treated with or without alkaline phosphatase (AP). Immunoblotting with antibodies specific to the phosphorylated CDK consensus sequence was positive for Ser- or Thr-phosphorylated FLAG-Kif26b (Figure 2C), and the signal was abolished by treatment with either the CDK inhibitor Roscovitine or AP. Because the antibodies against phosphorylated CDK substrates are also known to recognize the motifs phosphorylated by mitogen-activated protein kinases (MAPKs), we investigated whether MAPKs could phosphorylate Kif26b by transfecting HEK293 cells with FLAG-Kif26b and treating with U0126, an inhibitor of the MAPKs MEK1/2. The FLAG-tagged proteins were immunoprecipitated from the lysates with anti-FLAG beads. Immunoblotting analysis revealed that U0126 treatment did not affect the binding of anti-phospho-CDK substrate antibodies to FLAG-Kif26b (Figure S2A), suggesting that Kif26b is phosphorylated specifically by CDKs.

Thr-1859 and Ser-1962 in the C-terminal tail region of Kif26b met the criteria for possible CDK substrates (Figure 2A). In addition to these sites, there are another seven CDK consensus sequences in the remaining regions of Kif26b. Accordingly, we constructed Kif26b mutants in which these Ser/Thr residues were replaced with Ala and tested whether antibodies against phosphorylated CDK substrates could recognize these mutant proteins. Immunoprecipitation and immunoblotting analyses revealed that mutation of Thr-1859 or Ser-1962 (constructs Kif26b T1859A or Kif26b S1962A, respectively), but not other residues, of Kif26b significantly decreased its reactivity with antibodies against phosphorylated CDK substrates (Figure 2D). Moreover, an *in vitro* kinase assay demonstrated that purified CDK5 efficiently phosphorylated GST-Kif26b-C, but not GST, and that mutation of Thr-1859, Ser-1962, or both Thr-1859 and Ser-1962 of Kif26b to Ala impaired its phosphorylation by CDK5 (Figure S2B). These results suggest that CDKs directly phosphorylate Kif26b at both Thr-1859 and Ser-1962. To further confirm phosphorylation of Kif26b, antibodies specific for phosphorylated Thr-1859 or Ser-1962 were raised in rabbits immunized with synthetic peptides. The affinity-purified antibodies (anti-phospho-Thr1859 Kif26b and anti-phospho-Ser1962 Kif26b antibodies) recognized WT but not Ser/Thr-mutant FLAG-Kif26b immunoprecipitated with anti-FLAG beads (Figure S2C).

### Phosphorylation of Kif26b Promotes its Interaction with Nedd4

**Figure 3:**
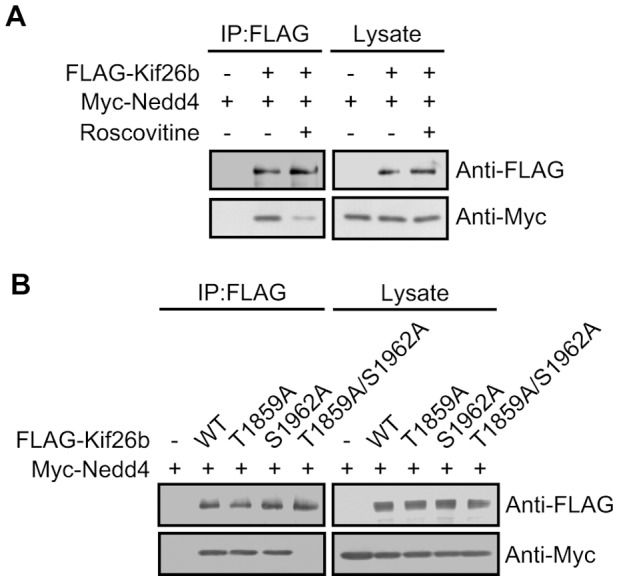
Kif26b interacts with Nedd4 in a phosphorylation-dependent manner. *A*. HEK293 cells were transfected with the indicated constructs. At 48 h after post-transfection, cells were treated for 6 h with DMSO (-) or Roscovitine (+; 20 µM) and then lysed, and the FLAG-tagged proteins were immunoprecipitated with anti-FLAG beads. The precipitates were analyzed by immunoblotting with the indicated antibodies. *B.* HEK293 cells were transfected with the indicated constructs. The FLAG-tagged proteins were immunoprecipitated from the cell lysates with anti-FLAG beads. The precipitates were analyzed by immunoblotting with the indicated antibodies.

Because the C-terminal tail region of Kif26b was required for the interaction between Kif26b and Nedd4 (Figure 1B and C), we next investigated whether phosphorylation of this region could affect the interaction of Kif26b with Nedd4. HEK293 cells were co-transfected with FLAG-Kif26b and Myc-Nedd4, treated with Roscovitine, and lysed, and FLAG-tagged proteins were immunoprecipitated from the lysates with anti-FLAG beads and subjected to immunoblotting. Roscovitine treatment clearly interfered with the interaction between Kif26b and Nedd4 (Figure 3A). We also tested the ability of Ala-substitution mutants of Kif26b to associate with Nedd4. Overexpression and immunoprecipitation analyses showed that Kif26b with both the T1859A and S1962A substitutions, but not with either alone, had significantly reduced affinity for Nedd4 (Figure 3B). These results suggest that phosphorylation of Kif26b at either Thr-1859 or Ser-1962 enhances the interaction between Kif26b and Nedd4.

### Kif26b is Polyubiquitinated by Nedd4

**Figure 4:**
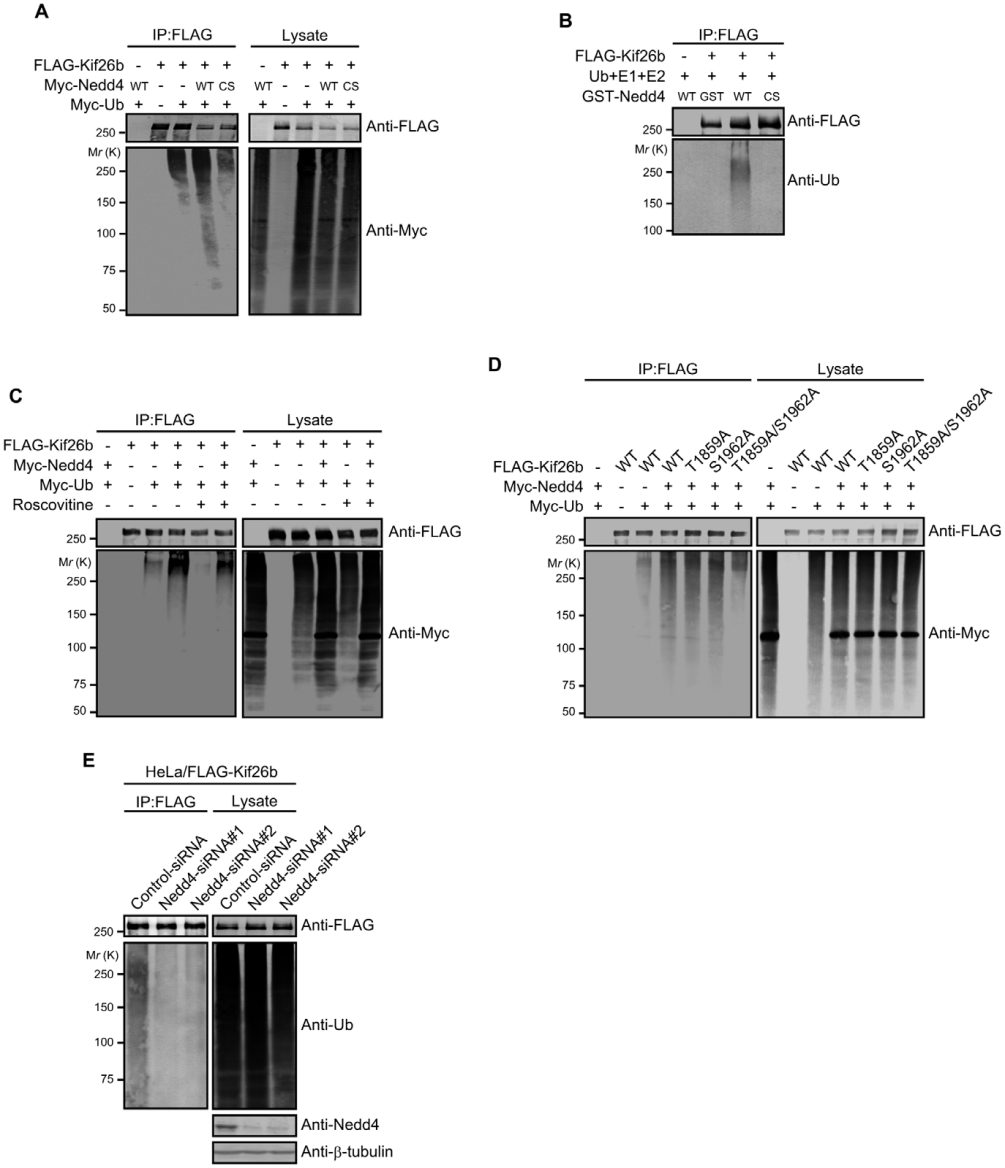
Ubiquitination of Kif26b is promoted by Nedd4. *A*. HEK293 cells were transfected with the indicated plasmids. At 48 h after post-transfection, the cells were treated for 8 h with MG132 (20 µM) and the FLAG-tagged proteins were then immunoprecipitated from the cell lysates with anti-FLAG beads. The precipitates were analyzed by immunoblotting with the indicated antibodies. *B*. HEK293 cells were transfected with FLAG-Kif26b. The FLAG-tagged proteins were immunoprecipitated from the cell lysates with anti-FLAG M2 beads, and the precipitates were used for *in vitro* ubiquitination assays with GST or GST-Nedd4 proteins (wild-type [WT] or catalytically inactive [CS]). The reaction mixtures were subjected to immunoblotting with the indicated antibodies. *C*. HEK293 cells were transfected with the indicated plasmids. At 48 h after post-transfection, the cells were pretreated for 3 h with DMSO or Roscovitine (20 µM) and then further treated for 8 h with MG132 (20 µM). The FLAG-tagged proteins were then immunoprecipitated from the cell lysates with anti-FLAG beads and subjected to immunoblotting with the indicated antibodies. *D*. HEK293 cells were transfected with the indicated plasmids. At 48 h after post-transfection, the cells were treated as in *A* and then the FLAG-tagged proteins were immunoprecipitated from the cell lysates with anti-FLAG beads and subjected to immunoblotting with the indicated antibodies. *E*. HeLa cells stably expressing FLAG-Kif26b were transfected with control or Nedd4-siRNAs. At 48 h after post-transfection, the cells were treated as in *A* and the FLAG-tagged proteins were then immunoprecipitated from the cell lysates with anti-FLAG beads. The precipitates were analyzed by immunoblotting with the indicated antibodies.

As Nedd4 is known to catalyze protein polyubiquitination, we examined whether Nedd4 mediated polyubiquitination of Kif26b. We expressed FLAG-Kif26b, Myc-Nedd4, and Myc-Ubiquitin (Ub) in HEK293 cells. At 48 h post-transfection, cells were treated for 8 h with the proteasome inhibitor MG132 and Kif26b was immunoprecipitated with anti-FLAG beads. We observed a smeared signal in the anti-FLAG bead precipitates of the lysates of cells expressing FLAG-Kif26b and Myc-Ub (Figure 4A). This smear signal was enhanced by further overexpression of Myc-Nedd4 WT but not Myc-Nedd4 CS. The smeared signal presumably represents polyubiquitinated Kif26b, as it persisted in the precipitates from the lysates treated with 2% SDS and boiled for 10 min to disrupt any noncovalent interactions between Kif26b and other proteins (Figure S3A *)*. This method has previously been reported to remove associated proteins from the immunoprecipitated protein of interest [24]. Furthermore, we also performed *in vitro* ubiquitination assays. FLAG-Kif26b was expressed in HEK293 cells and purified by immunoprecipitation with anti-FLAG beads as an ubiquitination substrate. Then, GST, GST-Nedd4 WT, or GST-Nedd4 CS purified from *E. coli* and ubiquitin, together with other E1 and E2 proteins, were added to initiate the ubiquitination reaction. The positive smeared signal was observed and was dependent on the catalytic activity of Nedd4 (Figure 4B). We also found that polyubiquitination of Kif26b involved Lys48 but not Lys63 of ubiquitin (Figure S3B). Lys48-linked polyubiquitin chains have been reported to target proteins for proteasomal degradation [25]. Therefore, as shown in Figure 1A with Nedd4 WT and CS, Kif26b appears to be not only a substrate for Nedd4 but also to be targeted by it for proteasomal degradation.

Phosphorylation of Kif26b regulated its interaction with Nedd4 (Figure 3). Therefore, we examined whether phosphorylation also affected polyubiquitination of Kif26b. Overexpression and immunoprecipitation analyses demonstrated that treatment with Roscovitine decreased the amount of polyubiquitinated Kif26b (Figure 4C). Furthermore, the Kif26b T1859A/S1962A mutant showed reduced polyubiquitination when co-expressed with Nedd4 (Figure 4D). These results suggest that Kif26b phosphorylation is important for its polyubiquitination by Nedd4.

In order to verify that endogenous Nedd4 also affected polyubiquitination of Kif26b, we treated HeLa/FLAG-Kif26b cells with siRNAs against Nedd4 and performed immunoprecipitation and immunoblotting analyses. Treatment with two siRNAs against Nedd4 reduced the amount of polyubiquitinated FLAG-Kif26b compared with a control siRNA treatment (Figure 4E), implying that endogenous Nedd4 mediate degradation of Kif26b via the ubiquitin-proteasome pathway.

### Disruption of Microtubules Promotes Phosphorylation and Polyubiquitination of Kif26b

We next addressed how upstream signaling induced the phosphorylation and polyubiquitination of Kif26b. Kif26b is expressed in the metanephric mesenchyme but is absent in the renal epithelia (described below). This suggests the possibility that epithelialization of the metanephric mesenchyme leads to degradation of Kif26b. Dramatic rearrangements to the microtubule-based cytoskeleton have been reported to occur during the formation of polarized epithelial cells [26], [27]. Therefore, we hypothesized that posttranslational modification of Kif26b could depend on the status of the microtubules.

**Figure 5:**
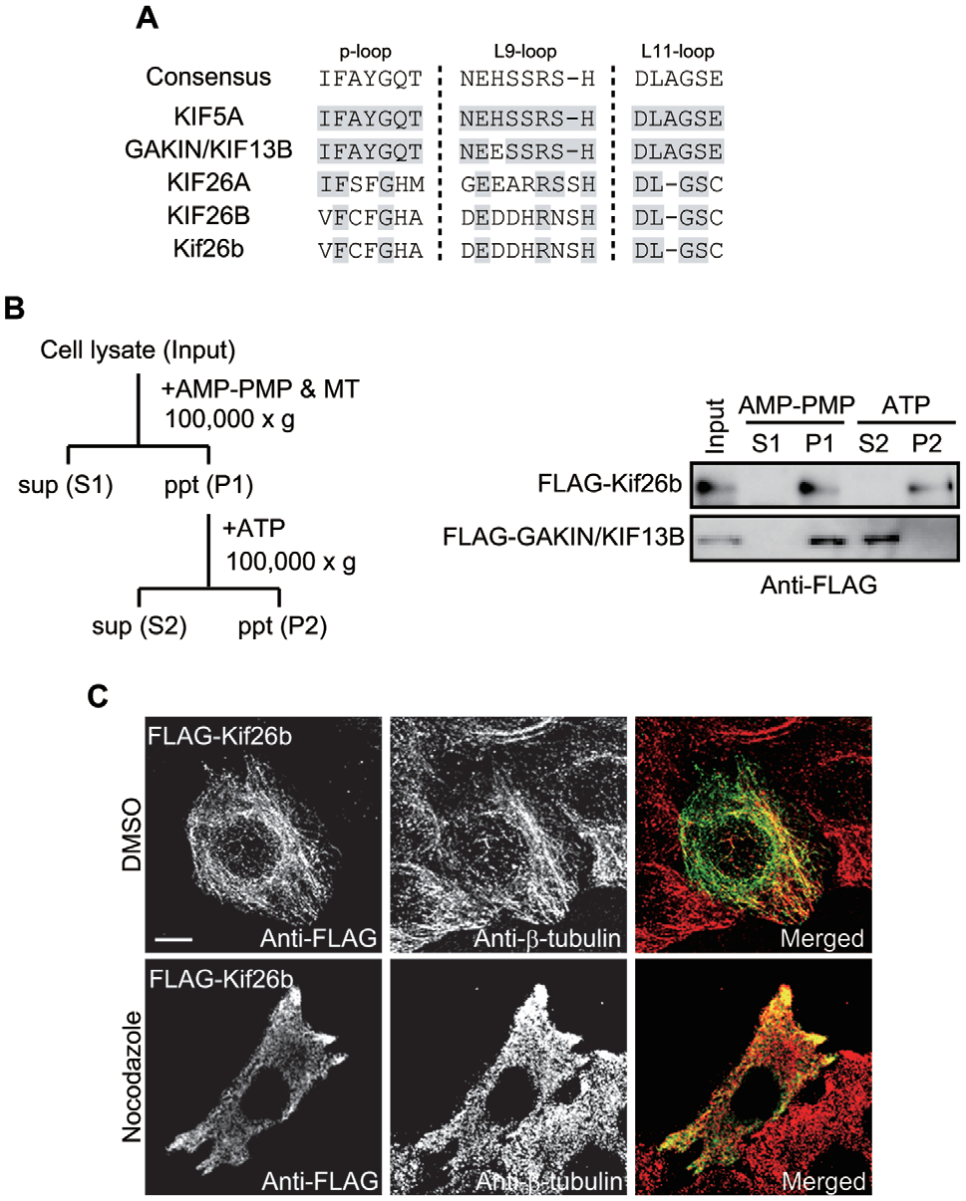
Kif26b is an unconventional kinesin. *A.* Alignment of the amino acid sequences of the motor domains of various KIFs. Human KIF5A, GAKIN/KIF13B, KIF26A, and KIF26B and mouse Kif26b are shown. Amino acids that correspond to the p-loop, L9-loop, and L11-loop consensus sequences are shown on a gray background. *B.* Schematic diagram of the microtubule-binding assay procedure (left panel). COS-7 cells were transfected with FLAG-Kif26b- or FLAG-GAKIN/KIF13B-expressing plasmids. At 48 h after post-transfection, the microtubule-binding assay was performed on the cell lysates and the precipitates were analyzed by immunoblotting with anti-FLAG antibody (right panel). *C.* HeLa cells stably expressing FLAG-Kif26b were treated for 1 h with DMSO or nocodazole (5 µM). The cells were then fixed and stained with anti-FLAG (green) and anti-β-tubulin (red) antibodies. Scale bar = 10 µm.

We first addressed whether Kif26b associated with microtubules. Kif26b, like Kif26a, is classified as a member of the Kinesin-11 family [6]. KIF26A has been reported to associate with microtubules but to lack microtubule-based motor activity [7]. As shown in Figure 5A, amino acid comparison with other KIFs demonstrates that KIF26B and Kif26b harbor neither the “IFAYGQT” nor “DLAGSE” motif, that is highly conserved in KIF motor domains and also differ in several amino acid sequences that function in ATP hydrolysis [6]. To determine whether Kif26b associated with microtubules and possessed ATPase-based motor activity, we performed an ATP-dependent microtubule-dissociation assay using FLAG-Kif26b expressed in COS7 cells. This experiment demonstrated that Kif26b constitutively associated with microtubules, while kinesin-like microtubule-based motor protein GAKIN/KIF13B dissociated from microtubules in response to ATP (Figure 5B). Immunostaining of HeLa/FLAG-Kif26b cells with anti-FLAG antibody showed a mesh-like structure that was obviously co-localized with microtubules (Figure 5C). In addition, nocodazole treatment disrupted the mesh-like distribution of Kif26b, suggesting that Kif26b localization was microtubule-based. Taken together, these results suggest that Kif26b associates with microtubules but lacks microtubule-based motor activity based on ATP hydrolysis.

**Figure 6:**
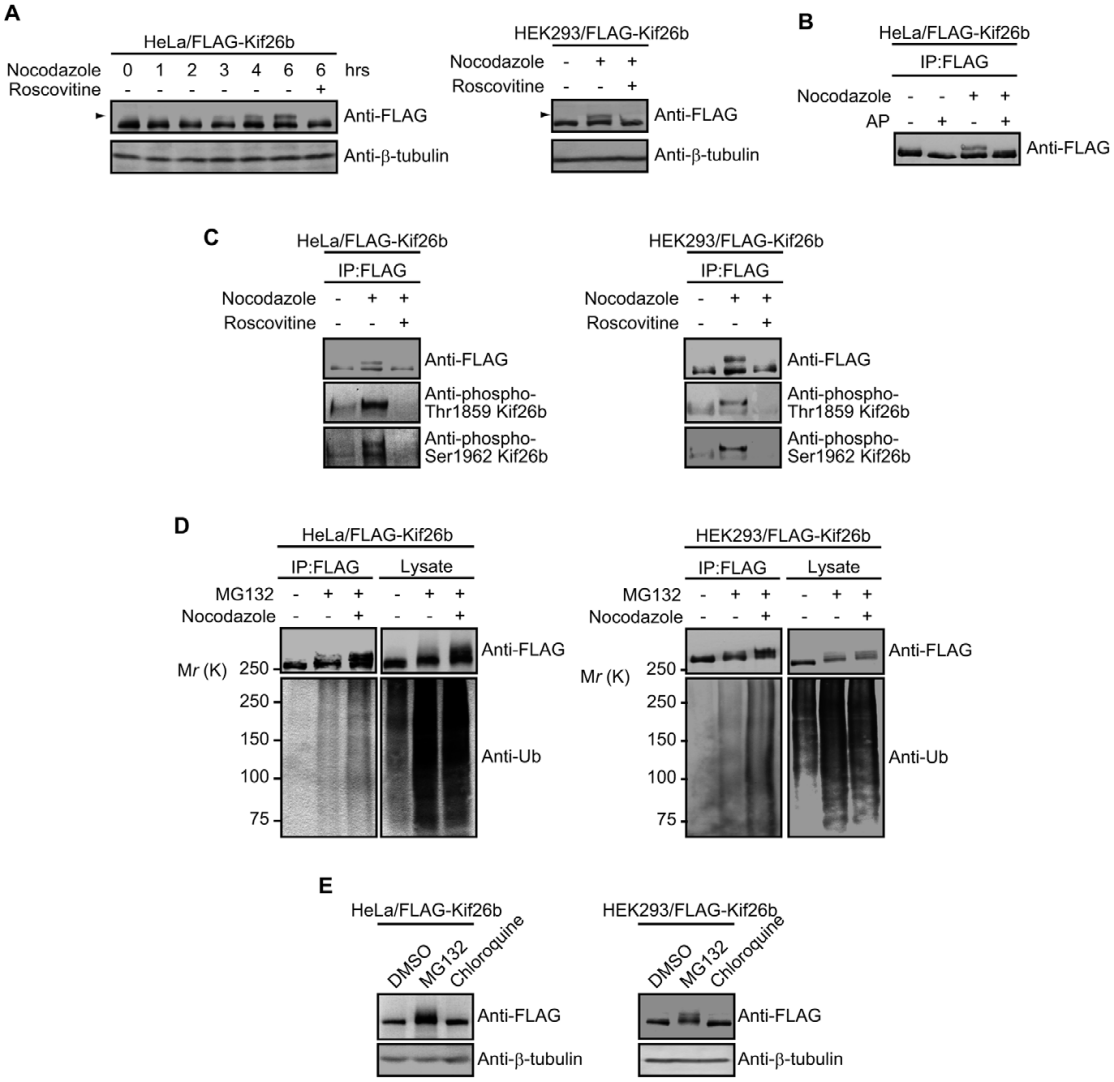
Phosphorylation and polyubiquitination of Kif26b are induced by disruption of microtubules. *A.* HeLa cells stably expressing FLAG-Kif26b were pretreated for 3 h with DMSO or Roscovitine (20 µM) and then treated for the indicated time with 5 µM nocodazole (left panel). HEK293 cells stably expressing FLAG-Kif26b were pretreated for 3 h with DMSO or Roscovitine (20 µM) and then treated for 6 h with 5 µM nocodazole (right panel). The lysates were subjected to immunoblotting with the indicated antibodies. *B.* HeLa cells stably expressing FLAG-Kif26b were treated for 6 h with nocodazole (5 µM), and then the FLAG-tagged proteins were immunoprecipitated from the cell lysates with anti-FLAG beads. The precipitates were subsequently treated with or without AP at 37°C for 1 h and analyzed by immunoblotting with anti-FLAG antibody. *C*. HeLa or HEK293 cells stably expressing FLAG-Kif26b (left and right panel, respectively) were treated as in *B,* and then the FLAG-tagged proteins were immunoprecipitated from the cell lysates with anti-FLAG beads. The precipitates were analyzed by immunoblotting with the indicated antibodies. *D*. HeLa or HEK293 cells stably expressing FLAG-Kif26b (left and right panel, respectively) were pretreated for 6 h with nocodazole and then further treated for 8 h with MG132 (20 µM). The FLAG-tagged proteins were immunoprecipitated from the lysates with anti-FLAG beads and subjected to immunoblotting with the indicated antibodies. *E*. HeLa or HEK293 cells stably expressing FLAG-Kif26b (left and right panel, respectively) were treated for 8 h with DMSO, MG132 (20 µM), or chloroquine (100 µM). Whole cell lysates were subjected to SDS-PAGE followed by immunoblotting with the indicated antibodies.

We next examined whether the phosphorylation of Kif26b was dependent on the microtubule status. When we treated HeLa/FLAG-Kif26b cells with nocodazole, we observed a significant shift of the FLAG-Kif26b signal toward the higher molecular weight area on SDS-PAGE in a time-course dependent manner (Figure 6A), and pretreatment with Roscovitine inhibited the mobility shift of Kif26b in response to nocodazole. We also found the mobility shift of FLAG-Kif26b induced by nocodazole treatment of HEK (human embryonic kidney) 293 cells stably expressing FLAG-Kif26b (HEK293/FLAG-Kif26b cells). Interestingly, the slower-migrating form of Kif26b disappeared upon AP treatment (Figure 6B), and, anti-phospho-Thr1859 Kif26b or anti-phospho-Ser1962 Kif26b antibody strongly recognized the slower-migrating form of Kif26b (Figure 6C). These results suggest that the mobility shift of Kif26 is attributable to phosphorylation by CDKs, which could be triggered by dissociation of Kif26b from microtubules.

We next addressed whether nocodazole treatment affected polyubiquitination of Kif26b. HeLa or HEK293/FLAG-Kif26b cells were pretreated for 6 h with DMSO or nocodazole and then treated for another 8 h with MG132. Immunoprecipitation and immunoblotting analyses showed that nocodazole treatment increased the polyubiquitination of Kif26b (Figure 6D). Furthermore, we observed a smeared and mobility-shifted FLAG-Kif26b band in response to MG132 treatment. This MG132-induced FLAG-Kif26b mobility shift was further enhanced by treatment with nocodazole. Interestingly, the FLAG-Kif26b mobility shift was not observed when we treated HeLa or HEK293/FLAG-Kif26b cells with the lysosomotropic drug chloroquine, which increases endosomal and lysosomal pH and impairs lysosomal function (Figure 6E). These results suggest that Kif26b dissociation from microtubules is followed by its phosphorylation by CDKs and polyubiquitination by Nedd4, resulting in its degradation via the ubiquitin-proteasomal pathway.

### Kif26b is Phosphorylated and Polyubiquitinated in the Developing Kidney

**Figure 7:**
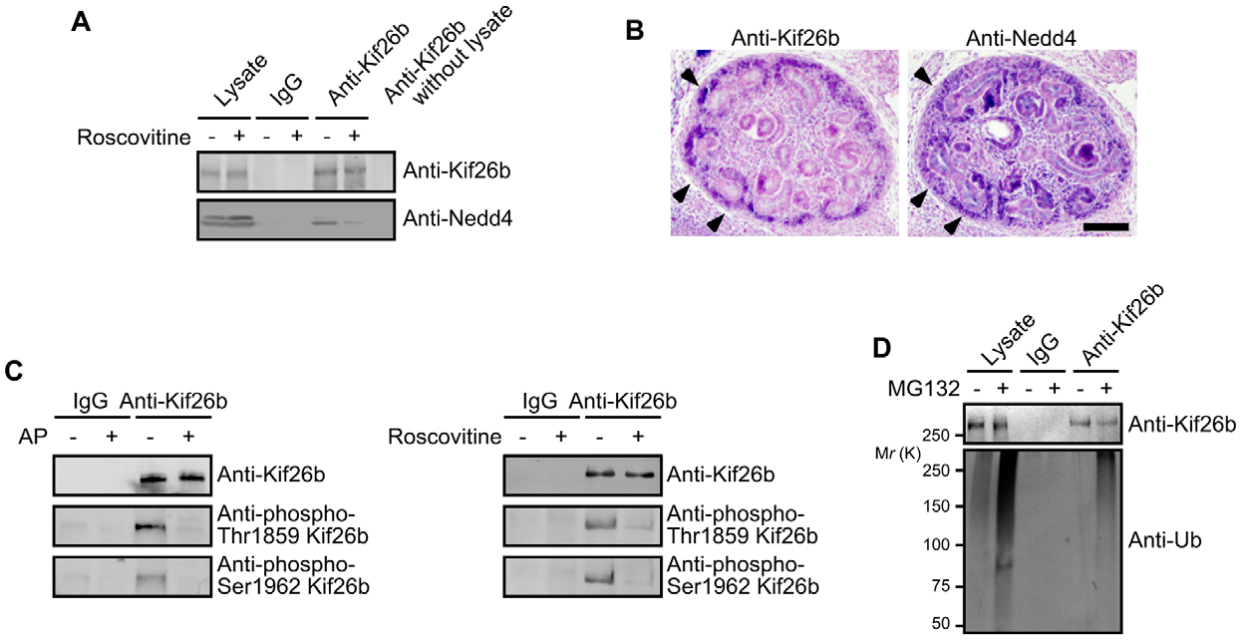
Phosphorylation and polyubiquitination of Kif26b in the developing kidney. *A*. E14.5 kidneys were cultured *in vitro* for 8 h with or without roscovitine (20 µM) (indicated as “+” or “−,” respectively). Kif26b was immunoprecipitated from the lysates with anti-Kif26b antibody and subjected to immunoblotting with the indicated antibodies. Immunoprecipitations with rabbit IgG and without lysate were performed as controls. *B*. Kidneys dissected from E14.5 embryos were stained with the indicated antibodies. Arrow heads indicate the metanephric mesenchyme. Scale bar = 10 µm. *C.* Kif26b was immunoprecipitated from E14.5 kidney lysates with anti-Kif26b antibody, and precipitates were treated with or without AP for 1 h at 37°C and analyzed by immunoblotting with the indicated antibodies (left panel). E14.5 kidneys were cultured *in vitro* for 8 h with or without roscovitine (20 µM) (indicated as “+” or “−,” respectively). Kif26b was immunoprecipitated and subjected to immunoblotting with the indicated antibodies (right panel). *D.* E14.5 kidneys were cultured *in vitro* for 8 h with or without MG132 (20 µM) (indicated as “+” or “−,” respectively). Kif26b was immunoprecipitated from the lysates with anti-Kif26b antibody and subjected to immunoblotting with the indicated antibodies.

As overexpression analyses had allowed us to determine how Kif26b is regulat̀ed by Nedd4, we next sought to verify the interaction between endogenous Kif26b and Nedd4 in the developing kidney. Immunoprecipitation from E14.5 kidney lysates showed that Nedd4 could be identified in anti-Kif26b but not control rabbit IgG precipitates (Figure 7A). The interaction was disturbed by treatment with roscovitine, suggesting that the *in vivo* complex formation between Kif26b and Nedd4 in the developing kidney is regulated by CDKs. Immunohistochemistry analyses of E14.5 kidneys showed that Kif26b was localized in the peripheral regions of the developing kidney, in which the metanephric mesenchyme containing nephron progenitors exist [28], but not in the epithelial components such as renal vesicles and comma-shaped bodies (Figure 7B). Nedd4 was also detected also in the metanephric mesenchyme as well as in the epithelialized structures (Figure 7B, arrow heads). *In situ* hybridization for Nedd4 showed an expression pattern similar to that shown by immunostaining (Figure S4). These data suggest the importance of Kif26b-Nedd4 interaction in the undifferentiated mesenchymal population.

Finally, we examined whether phosphorylation and polyubiquitination of endogenous Kif26b took place in the developing kidney. Kif26b was immunoprecipitated from E14.5 kidney lysates by anti-Kif26b antibody. Immunoblotting with anti-phosphorylated Kif26b antibodies showed obvious signals in the anti-Kif26 precipitates, which disappeared upon AP treatment or roscovitine treatment (Figure 7C). Furthermore, we also confirmed the interaction between Kif26b and CDK5 in E14.5 kidney by immunoprecipitation (Figure S5A). These results suggest CDKs can phosphorylate Kif26b in the developing kidney. We next examined Kif26b polyubiquitination in E14.5 embryonic kidneys cultured *in vitro* with or without MG132. We were able to observe the smeared signal by immunoblotting anti-Kif26b precipitates with anti-ubiquitin antibody (Figure 7D). Collectively, these results suggest that phosphorylation of Kif26b by CDKs in the metanephric mesenchyme enhances its polyubiquitination by Nedd4 and targets it for proteasomal degradation.

## Discussion

Genetic approaches have demonstrated the importance of several signaling pathways and transcription factors in kidney development. However, the mechanism by which the turnover of these crucial proteins is controlled has not been fully understood. In this study, we demonstrated that Kif26b in the developing kidney was phosphorylated by CDKs, interacted with Nedd4, and was polyubiquitinated. This suggests that posttranslational modification of Kif26b protein and its degradation by the ubiquitin-proteasome pathway are important in kidney development.

Although its highest expression level and kinase activity are detected in the nervous system, CDK5 is ubiquitously expressed in mammalian tissues [29]. *CDK5*-deficient mice are well known to show disruption of neuronal layering of many brain structures, such as the cerebral cortex and hippocampus, due to impaired migration of neuronal cells [30]. Several reports have demonstrated that CDK5 is also important for insulin secretion in pancreatic beta cells, differentiation of myogenic precursor cells, cell-matrix and cell-cell adhesion in lens epithelial cells, and survival of leukemia cells [31]–[34]. During kidney development, CDK5 is expressed in the metanephric mesenchyme and tubular structures involved in podocyte differentiation, proliferation, and morphology [35]. In regard to CDK2, its expression in the kidney is high during the embryonic period and slowly decreases after birth [36]. CDK2 is also expressed ubiquitously in many tissues, but its knockout mice are viable [37]. In this study, we presented evidence that CDKs phosphorylated Kif26b, suggesting an important role for CDKs, including CDK2 and CDK5, in the metanephric mesenchyme.

CDK2 and CDK5 show similar substrate specificity [29]. In particular, microtubule-associated proteins such as Tau, MAP2 and stathmin-like 2 have been identified as actual and potential targets by CDK2 and CDK5 [38]–[40]. We found that CDKs could phosphorylate both Thr-1859 and Ser-1962 on Kif26b (Figure 2B) and treatment with roscovitine resulted in a decreased phosphorylation of Kif26b in cultured cells and embryonic kidney explants (Figure 2C and 7C). We further examined the expression level of Kif26b in E17.5 kidney from *CDK5*-deficient mice [30]. However, immunoblotting analysis with anti-Kif26b antibody showed no significant difference in expression of Kif26b among wild type, *CDK5*-heterozygous or -homozygous mice (Figure S5B), suggesting that CDKs are functionally redundant in the regulation of Kif26b in the developing kidney.

One important issue that remains to be addressed is how phosphorylation of Kif26b is promoted. We found that phosphorylation of Kif26b by CDKs was enhanced by nocodazole treatment in HeLa and HEK293 cells (Figure 6A). CDK5 activation is regulated by its activator p35 or p39 [29]. It has been reported that microtubules, but not tubulin heterodimers, block the interaction between p35 and CDK5 by binding to p35, thereby inhibiting CDK5/p35 activity [41]. Therefore, disruption of microtubules could promote activation of CDKs in cultured cells. Because drastic reorganization of the cytoskeleton, including microtubules, occurs during epithelialization [26], [27], CDKs could also be activated during the transition from the metanephric mesenchyme to the epithelium, thereby phosphorylating Kif26b. Activation of canonical Wnt signaling is known to induce the mesenchymal to epithelial transition [42]. However, treating HeLa or HEK293/FLAG-Kif26b cells with Wnt3a-conditioned medium did not significantly increase phosphorylation of Kif26b as determined by immunoblotting with anti-phospho-Thr1859 or anti-phospho-Ser1962 Kif26b antibodies (data not shown). Therefore, we consider that epithelialization of the metanephric mesenchyme itself, but not inductive signals for epithelialization, could promote phosphorylation of Kif26b that leads to its polyubiquitination and degradation through the proteasomal pathway.

Ubiquitination is a key regulatory process that determines the fate of a protein and induces various cellular events including protein degradation, endocytosis, and transmembrane protein sorting and trafficking [43]. This modification is catalyzed by two major classes of E3 ubiquitin ligases, HECT domain-containing and RING domain-containing E3 ligases [44], [45]. Nedd4 and Nedd4-2 belong to the WW domain-containing HECT E3 ubiquitin ligase subfamily [18]. Several studies have demonstrated the importance of ubiquitination by Nedd4 in proteasomal degradation of many proteins, including CNrasGEF [22], sprouty2 [24], c-Cbl [46], and ErbB4 [47]. Nedd4 is therefore involved in regulating several signaling pathways, with important implications for animal development. Indeed, disruption of *Nedd4* in mice causes embryonic lethality at mid- to late gestation. *Nedd4*-knockout embryos are smaller than wild-type littermates, and *Nedd4*-null mouse embryonic fibroblasts have reduced mitogenic activity [48], [49]. Recent studies have also reported that Nedd4 is essential for dendrite arborization and heart development [50], [51]. Further examination warrants the role of Nedd4 *in vivo* in kidney development. Several studies have reported that Nedd4-2 interacts with epithelial Na^+^ channel (ENaC) and mediates its ubiquitination, promoting its endocytosis from the plasma membrane and degradation [52], [53]. This mechanism prevents excess Na^+^ uptake in kidney epithelial cells, and defects in this regulatory pathway contribute to the pathogenesis of certain forms of hypertension such as Liddle syndrome, a hereditary hypertension that shows increased activity and retention of ENaC at the plasma membrane [54]. These data suggest that Nedd4 family proteins are important in both physiological and pathological events in the kidney.

The proteasomal degradation of Kif26b could contribute to its elimination from differentiated structures such as renal vesicles and comma-shaped bodies. However, the mechanism by which transcription of *Kif26b* is attenuated in the epithelialized structures remains unclear. Once the metanephric mesenchyme forms renal vesicles upon the ureteric bud in response to activation of canonical Wnt signals, *Kif26b* transcription is rapidly downregulated, while *Sall1* expression is sustained in this and subsequent stages of epithelial differentiation [9]. Therefore, decreased transcription of *Kif26b* cannot be explained solely by decreased expression of *Sall1*. Several studies have demonstrated that Sall1 acts as a transcriptional repressor. Sall1 associates with histone deacetylases, deacetylase corepressor complex, and several components of chromatin remodeling complexes [55]–[57]. These associations suggest the possibility that Sall1 negatively regulates *Kif26b*-transcription following the initiation of epithelialization induced by canonical Wnt signaling. It will be interesting to investigate the mechanism of transcriptional attenuation of *Kif26b*.

While we found phosphorylation and polyubiquitination of endogenous Kif26b, the physiological significance of these phenomena in kidney development awaits further studies *in vivo*. It would be necessary to generate a mouse strain that retains the phosphorylation-resistant Kif26b to see whether mis-regulation of Kif26b affects kidney formation. In addition, the physiological role of Nedd4 should be examined *in vivo*. We performed knockdown analysis of Nedd4 in kidney explants from E12.5 embryos using in vivo morpholino oligonucleotides (MO), which has been shown to be effective in the embryonic kidney [58]. The treatment with Nedd4-MO resulted in a slight accumulation of Kif26b after 24 h compared with control-MO, but this increase was not significant and we were also unable to rule out the possibility of off-target effects of the MO (data not shown). Therefore generation of kidney-specific mutant mice of Nedd4 would be required, though this molecule could affect multiple targets in addition to Kif26b.

In this study, we have identified the molecular mechanism by which Kif26b is degraded. Kif26b appears to be tightly regulated at both the transcriptional and posttranslational levels. Spatiotemporal regulation of Kif26b would be important for mesenchymal cells, which is indispensable for proper kidney formation.

## Supporting Information

Figure S1 **Identification of Nedd4 as a Kif26b-interacting protein.** The lysates from newborn kidneys were subjected to pull-down assay with recombinant GST-tagged C-terminal region of Kif26b. The precipitates were separated by SDS-PAGE, followed by silver staining. The arrowheads indicate proteins that were identified by mass spectrometry (top, NMHC IIB; bottom, Nedd4).
(TIF)

Figure S2 **Kif26b is phosphorylated by CDKs.** *A*. HEK293 cells were transfected with FLAG-Kif26b expressing plasmid. At 48 hrs after transfection, cells were treated with DMSO, Roscovitine (20 µM) or U0126 (20 µM) for 6 hrs. The lysates were subjected to immunoprecipitation with anti-FLAG beads. The precipitants were analyzed by immunoblotting with the indicated antibodies. *B*. GST, GST-Kif26b-C, GST-Kif26b-C T1859A, GST-Kif26b-C S1962A, or GST-Kif26b-C T1859A/S1962A was incubated with or without recombinant His-tagged CDK5, and a kinase assay was performed. Proteins were separated by SDS-PAGE and detected by Coomassie Brilliant Blue (CBB) staining or immunoblotting with the indicated antibodies. *C*. HEK293 cells were transfected with the indicated plasmids. At 48 hrs after transfection, cell lysates were subjected to immunoprecipitation with anti-FLAG beads, followed by immunoblotting with the indicated antibodies.
(TIF)

Figure S3 **Kif26b is polyubiquitinated via Lys48 on ubiquitin.** *A*. HEK293 cells were transfected with FLAG-Kif26b. At 48 hrs after transfection, cells were treated with MG132 (20 µM) for 8 hrs and lysed with lysis buffer in the presence or absence of 2% SDS. The lysate containing SDS was boiled for 10 min at 95°C, and then diluted 15 times with lysis buffer. Immunoprecipitation was performed with anti-FLAG M2 beads and the precipitants were subjected to SDS-PAGE, followed by immunoblotting with the indicated antibodies. *B*. HEK293 cells were transfected with Myc-tagged WT, K48R, K63R and 0K ubiquitin constructs along with FLAG-Kif26b. After 48 hrs, cells were treated with MG132 (20 µM) for 8 hrs, followed by immunoprecipitation with anti-FLAG M2 beads. The precipitates were subjected to SDS-PAGE and immunoblotting with the indicated antibodies.
(TIF)

Figure S4 **Nedd4 is expressed in developing kidney.** *In situ* hybridization showed Nedd4 was expressed in all components of developing kidney such as the metanephric mesenchymes, comma-shaped bodies and the ureteric epithelia. No signal was observed with the sense control probe. Scale bar, 100 µm.
(TIF)

Figure S5 **Interaction of Kif26b with CDK5 and expression of Kif26b in the developing kidney.** *A*. Kif26b was immunoprecipitated from E14.5 kidney lysates with anti-Kif26b antibody, and precipitates were analyzed by immunoblotting with the indicated antibodies. *B*. Kidneys from E17.5 CDK5 mutant embryos were lysed with sample buffer and then separated by SDS-PAGE followed by immunoblotting with the indicated antibodies.
(TIF)
