# Supplementary figures and images for: Phosphorylation of Kif26b Promotes Its Polyubiquitination and Subsequent Proteasomal Degradation during Kidney Development
Source: PLoS One. 2012 Jun 29;7(6):e39714. doi: 10.1371/journal.pone.0039714 (PMC3387196; doi:10.1371/journal.pone.0039714)

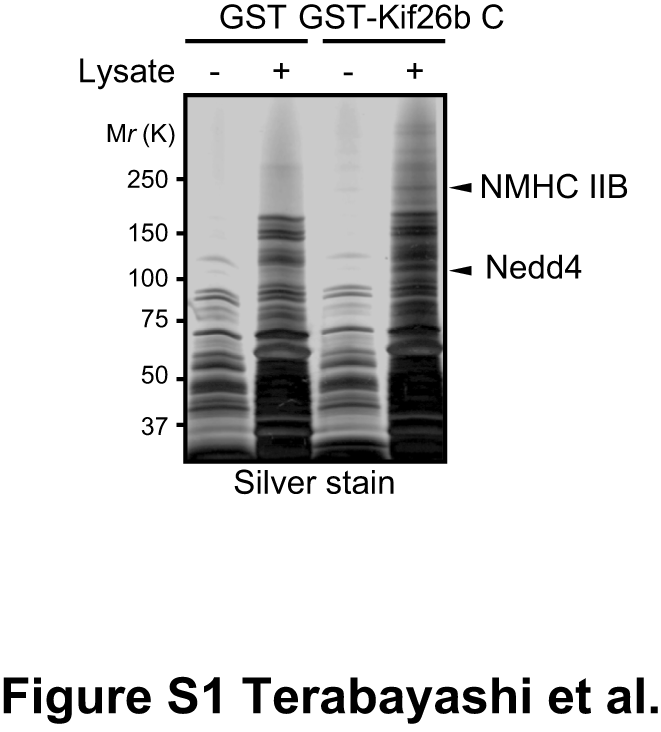

Supplement: Figure S1 — Identification of Nedd4 as a Kif26b-interacting protein. The lysates from newborn kidneys were subjected to pull-down assay with recombinant GST-tagged C-terminal region of Kif26b. The precipitates were separated by SDS-PAGE, followed by silver staining. The arrowheads indicate proteins that were identified by mass spectrometry (top, NMHC IIB; bottom, Nedd4). (TIF) [file pone.0039714.s001.tif]

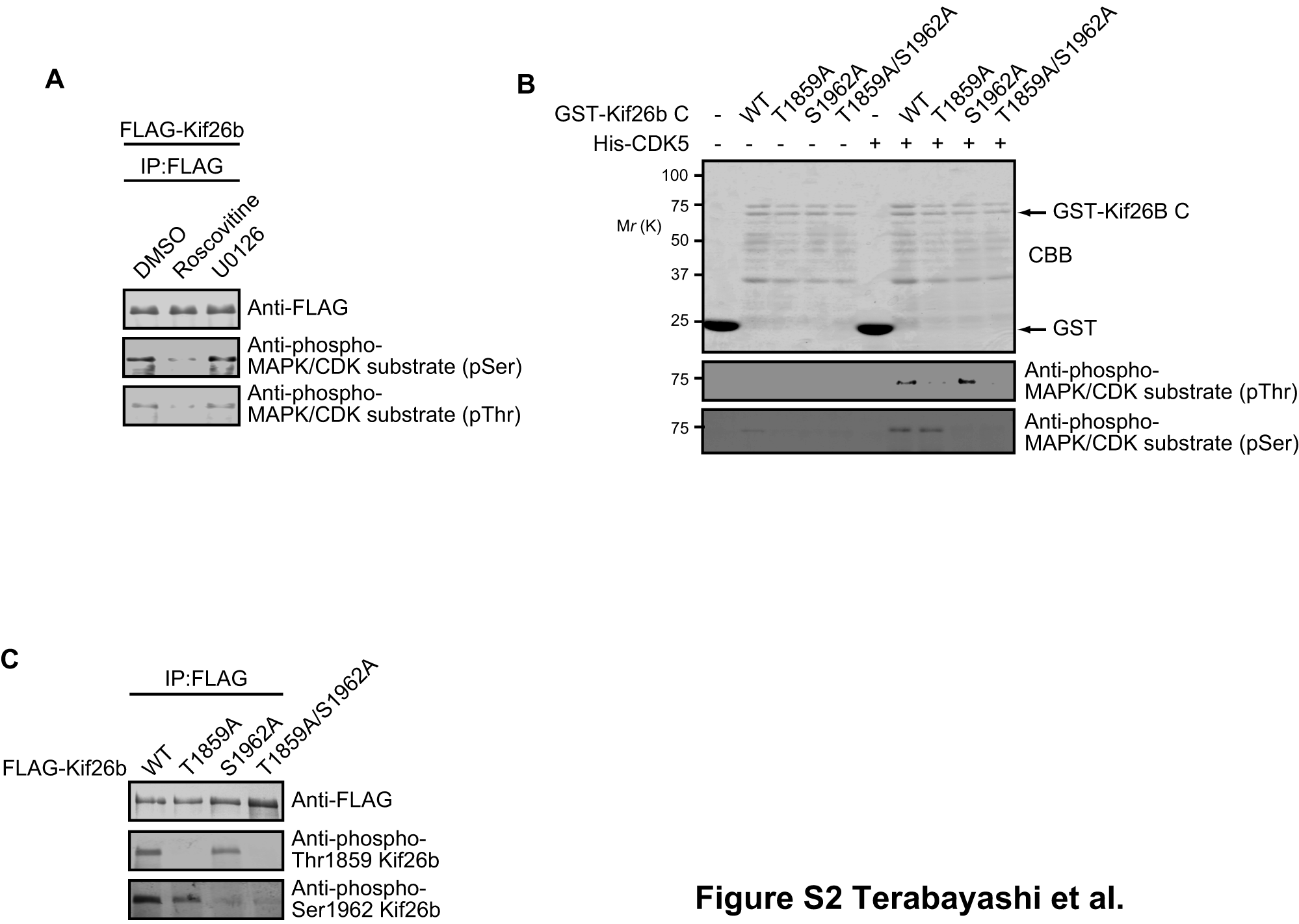

Supplement: Figure S2 — Kif26b is phosphorylated by CDKs. A. HEK293 cells were transfected with FLAG-Kif26b expressing plasmid. At 48 hrs after transfection, cells were treated with DMSO, Roscovitine (20 µM) or U0126 (20 µM) for 6 hrs. The lysates were subjected to immunoprecipitation with anti-FLAG beads. The precipitants were analyzed by immunoblotting with the indicated antibodies. B. GST, GST-Kif26b-C, GST-Kif26b-C T1859A, GST-Kif26b-C S1962A, or GST-Kif26b-C T1859A/S1962A was incubated with or without recombinant His-tagged CDK5, and a kinase assay was performed. Proteins were separated by SDS-PAGE and detected by Coomassie Brilliant Blue (CBB) staining or immunoblotting with the indicated antibodies. C. HEK293 cells were transfected with the indicated plasmids. At 48 hrs after transfection, cell lysates were subjected to immunoprecipitation with anti-FLAG beads, followed by immunoblotting with the indicated antibodies. (TIF) [file pone.0039714.s002.tif]

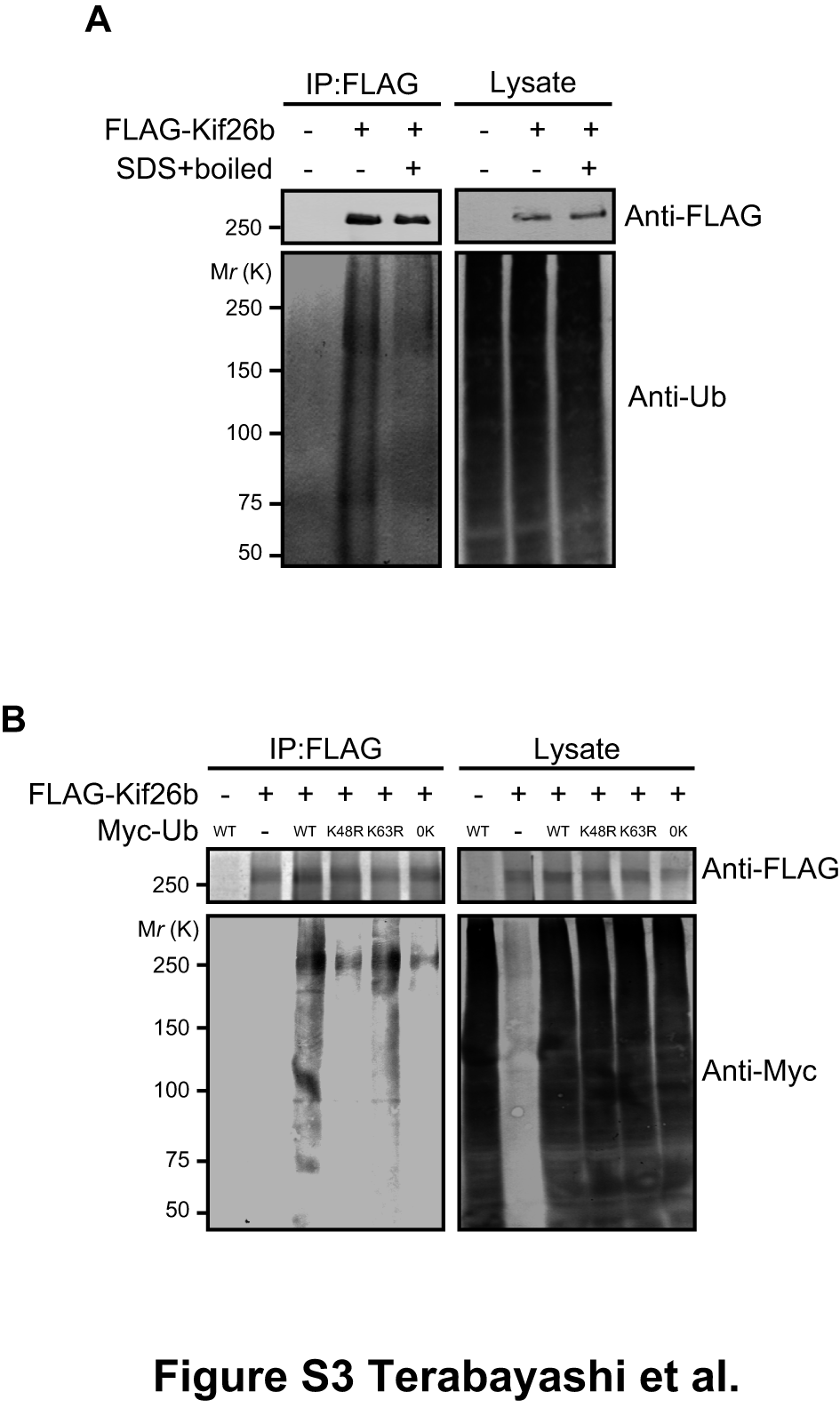

Supplement: Figure S3 — Kif26b is polyubiquitinated via Lys48 on ubiquitin. A. HEK293 cells were transfected with FLAG-Kif26b. At 48 hrs after transfection, cells were treated with MG132 (20 µM) for 8 hrs and lysed with lysis buffer in the presence or absence of 2% SDS. The lysate containing SDS was boiled for 10 min at 95°C, and then diluted 15 times with lysis buffer. Immunoprecipitation was performed with anti-FLAG M2 beads and the precipitants were subjected to SDS-PAGE, followed by immunoblotting with the indicated antibodies. B. HEK293 cells were transfected with Myc-tagged WT, K48R, K63R and 0K ubiquitin constructs along with FLAG-Kif26b. After 48 hrs, cells were treated with MG132 (20 µM) for 8 hrs, followed by immunoprecipitation with anti-FLAG M2 beads. The precipitates were subjected to SDS-PAGE and immunoblotting with the indicated antibodies. (TIF) [file pone.0039714.s003.tif]

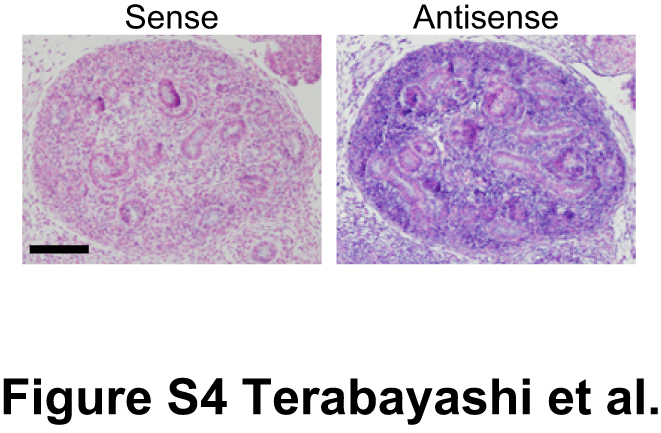

Supplement: Figure S4 — Nedd4 is expressed in developing kidney. In situ hybridization showed Nedd4 was expressed in all components of developing kidney such as the metanephric mesenchymes, comma-shaped bodies and the ureteric epithelia. No signal was observed with the sense control probe. Scale bar, 100 µm. (TIF) [file pone.0039714.s004.tif]

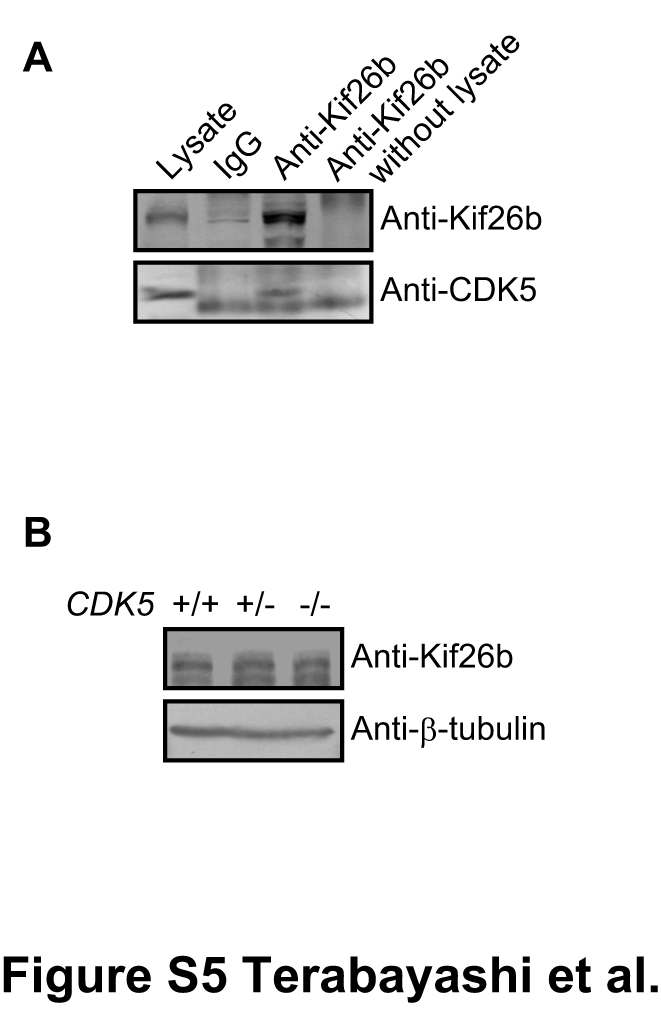

Supplement: Figure S5 — Interaction of Kif26b with CDK5 and expression of Kif26b in the developing kidney. A. Kif26b was immunoprecipitated from E14.5 kidney lysates with anti-Kif26b antibody, and precipitates were analyzed by immunoblotting with the indicated antibodies. B. Kidneys from E17.5 CDK5 mutant embryos were lysed with sample buffer and then separated by SDS-PAGE followed by immunoblotting with the indicated antibodies. (TIF) [file pone.0039714.s005.tif]
